# The investigation of stress in freestanding GaN crystals grown from Si substrates by HVPE

**DOI:** 10.1038/s41598-017-08905-y

**Published:** 2017-08-17

**Authors:** Moonsang Lee, Dmitry Mikulik, Mino Yang, Sungsoo Park

**Affiliations:** 1 Korea Basic Science Institute, Daejeon, 169-148 Republic of Korea; 2 Ecole Polytechnique Fédérale de Lausanne, Laboratory of Semiconductor Materials, Lausanne, 1015 Switzerland; 3 Korea Basic Science Institute, Seoul Center, Seoul, 5 Republic of Korea; 4 Jeonju University, Department of Science Education, Jeonju, 303 Republic of Korea; 5 Jeonju University, Analytical Laboratory of Advanced Ferroelectric Crystals, Jeonju, 303 Republic of Korea

## Abstract

We investigate the stress evolution of 400 µm-thick freestanding GaN crystals grown from Si substrates by hydride vapour phase epitaxy (HVPE) and the *in situ* removal of Si substrates. The stress generated in growing GaN can be tuned by varying the thickness of the MOCVD AlGaN/AlN buffer layers. Micro Raman analysis shows the presence of slight tensile stress in the freestanding GaN crystals and no stress accumulation in HVPE GaN layers during the growth. Additionally, it is demonstrated that the residual tensile stress in HVPE GaN is caused only by elastic stress arising from the crystal quality difference between Ga- and N-face GaN. TEM analysis revealed that the dislocations in freestanding GaN crystals have high inclination angles that are attributed to the stress relaxation of the crystals. We believe that the understanding and characterization on the structural properties of the freestanding GaN crystals will help us to use these crystals for high-performance opto-electronic devices.

## Introduction

Gallium nitride (GaN) is one of the most promising materials for optoelectronic devices such as laser diodes, ultra violet detectors, light-emitting diodes (LEDs), and power electronics^1–3^. Owing to the absence of natural nitrides, heteroepitaxial growth on foreign substrates is inevitable for the growth of GaN layers. This, however, leads to the generation of high dislocation density (10^7^–109/cm^2^) in the grown GaN layers, grading the optical and electrical performances of GaN-based devices^4–6^. As an alternative, the freestanding GaN is introduced.

Even if current commercial freestanding GaN substrates have been grown on Al_2_O_3_ or GaAs substrates by HVPE, obstacles such as size limitations (<6 inch diameter) and high production costs have to be overcome for the commercial success of freestanding GaN wafers^7^. The use of Si wafers as the heteroepitaxy substrates can be a feasible solution for achieving freestanding GaN^8^. However, the growth of GaN on Si substrates has been considered impossible because of the large lattice mismatch (16.9%) and the tensile stress on the GaN layers owing to the difference between the thermal expansion coefficients of GaN and Si, which results in the formation of cracks and a high dislocation density after the growth of GaN^9^. In addition, the meltback or the formation of a Ga-Si alloy by the reaction of Ga and Si, owing to the out-diffusion of Si atoms into GaN layers, restricts the adoption of Si substrates for obtaining freestanding GaN wafers^10^. To overcome these problems, various approaches have been studied. Wang *et. al*. demonstrated 30-nm-thick GaN epitaxial films on Si substrates using an Al buffer layer by using the combination of molecular beam epitaxy (MBE) and pulsed laser deposition (PLD)^8^. Bessolov *et. al*. achieved the growth of a 15-µm-thick HVPE GaN on a Si substrate using SiC nano buffer layer^11^. Despite these significant studies, until recently there has been no practical solution for developing a crack-free freestanding GaN from a Si substrate.

Recently, we briefly reported a crack-free freestanding GaN with a diameter and thickness of 2 inches and 400 µm, respectively, grown from Si wafers via the *in situ* removal of substrates by HVPE^12^. In this report, we characterize the stress evolution of the freestanding GaN crystals grown from Si substrates by HVPE. Understanding, determining, and characterizing the structural properties of the freestanding GaN crystals grown from Si substrates provides useful information, guiding the optimization of the performance of devices based on freestanding GaN crystals.

## Experimental

The growth experiments were performed using a modified HVPE reactor as illustrated in Fig. S1. To realize a new concept in the growth of freestanding GaN crystals from Si substrates, a HVPE reactor was modified into the configuration with a special HCl gas nozzle and susceptor to remove the Si substrates after growth at high temperature. To etch Si substrates at high temperature, an additional HCl gas nozzle was added to the bottom of the support to hold the quartz susceptor where HCl gas flowed through. With the exception of the additional HCl gas channel, the configuration of the modified HVPE system was similar to that used for a conventional vertical-type HVPE. A detailed description of the modified HVPE reactor is provided in the electronic supplementary information.

**Figure 1:**
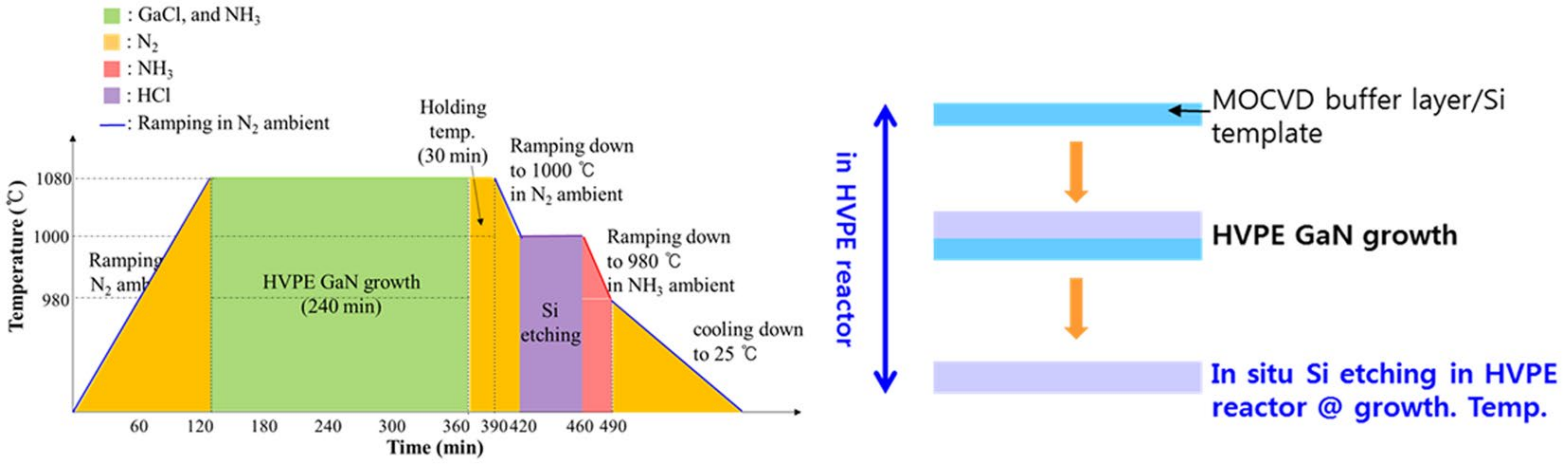
Schematic illustrations of (**a**) the growth procedure of freestanding GaN crystals grown on Si substrates and (**b**) its sequence in HVPE reactor.

The schematic diagram of the growth sequence of freestanding GaN crystals is detailed in Fig. 1. A 2-inch (111) Si wafer was used as a substrate for the growth of the GaN layer. The Si substrates were immersed in H_2_SO_4_: H_2_O_2_: H_2_O(3:1:1) (SPM) and HF (6%) solutions to obtain a hydrogen-terminated surface and an native oxide-free Si substrate, respectively. The growth of buffer layers was performed at 1000 °C in a commercial metal organic chemical vapour deposition (MOCVD) reactor (Veeco). An AlN buffer layer with the thickness ranging from 100 nm to 250 nm and an Al_0.4_Ga_0.6_N transition layer with the thickness ranging from 500 nm to 1.3 µm were grown sequentially on the Si substrate to prevent meltback and to release the stress due to the lattice mismatch between the Si substrate and GaN. For the AlN layer and Al_0.4_Ga_0.6_N growth, trimethylaluminium (TMAl), trimethylgallium (TMGa) and NH_3_ were employed as aluminium (Al), gallium (Ga) and nitrogen (N) sources, respectively, and hydrogen (H_2_) was used as the carrier gas at the pressure of 100 Torr. Next, a 1-µm-thick un-doped GaN (u-GaN) layer with a full width at half maximum (FWHM) of 250 arcsec in the X-ray rocking curve was grown at the same temperature using MOCVD. Subsequently, a 400-µm-thick GaN layer was grown on the MOCVD u-GaN/Al_0.4_Ga_0.6_N/AlN/Si template under atmospheric pressure and 1080 °C using a HVPE system capable of etching the Si substrate. For HVPE growth, HCl (flow rate: 40 sccm) was prereacted with liquid Ga to form the GaCl gas. Then, GaCl was transported to the growth zone where it reacted with NH_3_, generating the GaN layer. Here, N_2_ was used as the carrier gas, and the V/III ratio was equal to 20. Then, the Si substrate was etched by using HCl gas at approximately 1000 °C before the HVPE reactor was cooled to room temperature. Finally, the freestanding GaN crystals were successfully obtained. The structures of the MOCVD buffer layer templates and the growth procedures are described in detail in Table 1 and Fig. S2.

**Table 1:** Characteristics of the thickness of MOCVD buffer layers used in this study.

No.	AlN (nm)	Al_0.4_Ga_0.6_N (nm)	u-GaN (nm)
I	100	500	1000
II	180	920	
III	250	1250	

The structural properties of the freestanding GaN crystals were analysed using transmission electron microscopy and scanning electron microscopy. Energy dispersive X-ray spectroscopy (EDX) analysis was used to evaluate the chemical composition of the region of the meltback reaction in the freestanding GaN crystals. For the evaluation of the stress on the freestanding GaN crystals, Raman spectra of the freestanding GaN crystals were measured at room temperature, with the laser incidence on the surface and cross section of the freestanding GaN crystals. The Raman system used an argon ion laser with 514.5 nm output for excitation. Additionally, a Jasco NR100 spectrometer with the focal length of 1 m and a holographic grating with 1800 grooves/mm was used.

## Results and Discussion

**Figure 2:**
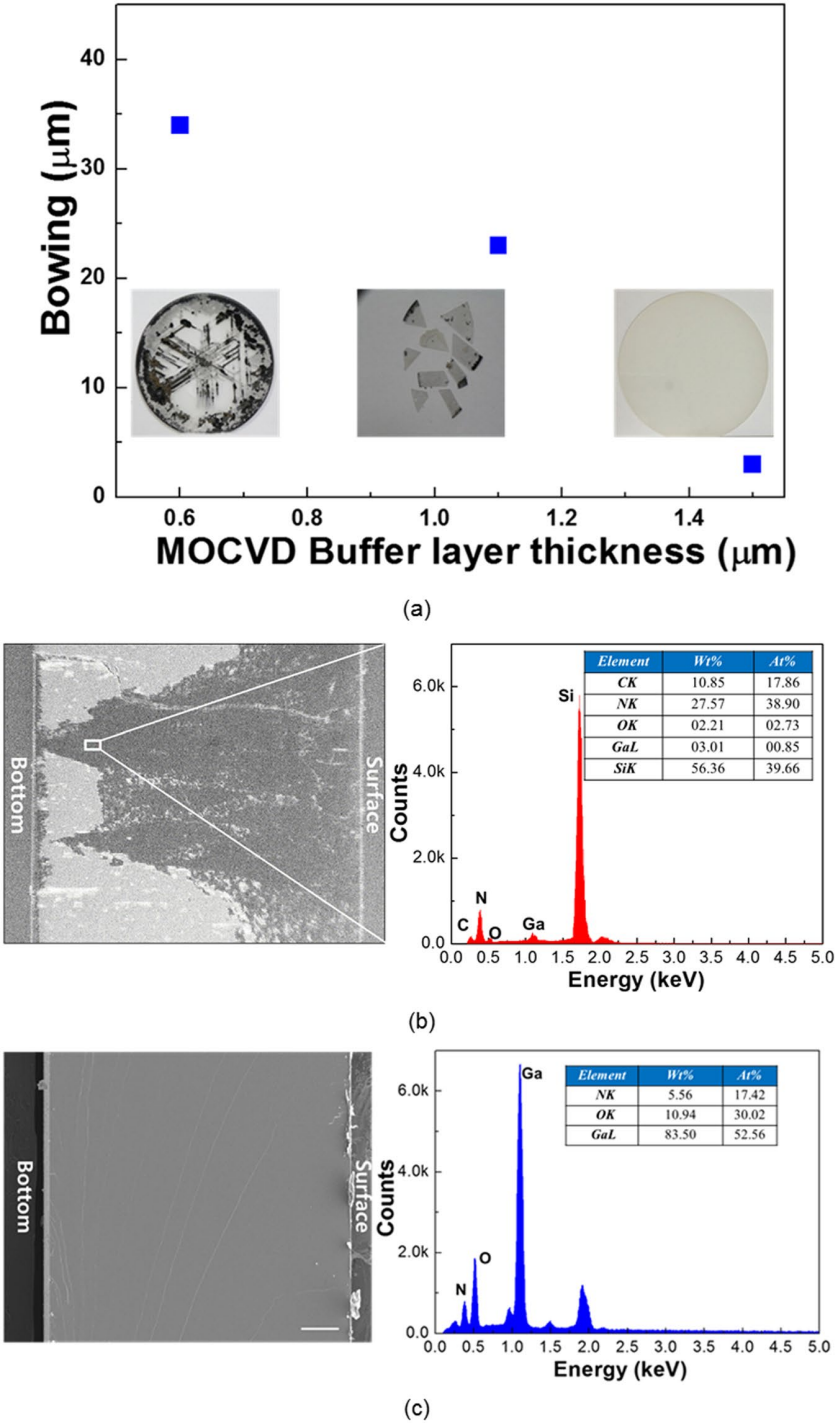
(**a**) MOCVD buffer layer thickness versus bowing of templates. The images represent the growth dependence on the thickness of the MOCVD template. Cross-sectional SEM images of the freestanding GaN grown on the MOCVD buffer layer with the thicknesses of (**b**) 0.6 µm, and (**c**) 1.5 µm. All scale bars represent 50 µm. EDX investigations show the presence of meltback in the freestanding GaN layers via the analysis of their chemical composition.

Figure 2(a) shows the images of the freestanding GaN crystals with different thicknesses of the MOCVD buffer layers, except for the thickness of the u-GaN layer.

Note that the value of bowing in the MOCVD buffer layer/Si templates decreases with increasing MOCVD buffer layer thickness, implying the increase in the compressive stress in MOCVD buffer layers^13^. When using MOCVD buffer layer with the thickness of 0.6 µm, freestanding GaN always had the meltback area. To confirm this, we performed EDX analysis of that area as shown in Fig. 2(b). EDX element analysis revealed the presence of a large Si content of approximately 40% atomic concentration in the dark area as well as a strong depletion of Ga up to detection levels (below 1%). These results suggest that a massive Si migration from the substrate occurs during the high-temperature growth process. Si migration from the substrate to the surface had been previously observed in GaN layers grown on Si and was related to the columnar structure of heteroepitaxial GaN layers and to the presence of the grain boundaries^14^. Pipe diffusion through threading dislocations may also enhance Si migration, which, owing to the strong reactivity of Ga and Si at high temperatures, gives rise to the meltback etching effect. We can clearly observe round melt-back areas that can be originated from point defects such as grain boundaries or threading dislocation. The crack-shape melt-back area can be described by the crack formation at the interface between GaN and Si during the high-temperature process. Meanwhile, with increasing thickness of the MOCVD buffer layers, the area of the meltback effect in freestanding GaN could be decreased. Finally, the crack-free freestanding GaN without meltback could be obtained using a MOCVD buffer layer with the thickness of 1.5 µm. The results of the EDX analysis shown in Fig. 2(c) confirmed that there are no Si atoms in the freestanding GaN layers, indicating that no meltback phenomena occurred when the freestanding GaN was grown using a MOCVD buffer layer with the thickness of 1.5 µm. This implies that the presence of the MOCVD buffer layers successfully contributes to the strain compensation for growing HVPE GaN layers^13, 15^. A 400-µm-thick freestanding GaN with a diameter of 2 inches was grown from a Si substrate with slightly concave bowing of 20 µm/2 inches, implying the presence of tensile stress in the freestanding GaN layers^16^. This is attributed to the difference in the crystal quality between the Ga- and N-faces of the freestanding GaN^17^. The FWHM for the (0002) X-ray rocking curve of the freestanding GaN was evaluated as 62 arcsec. (See Fig. S4) This value is comparable to or even better than those in GaN layers grown on other foreign substrates as reported elsewhere^4, 18^. Despite the high crystal quality of the freestanding GaN grown from Si substrates, no cracks and no meltback were observed in the GaN layers. We believe that the stress relaxation via sufficient stress compensation enables the growth of crack-free freestanding GaN with high crystal quality on Si substrates.

**Figure 3:**
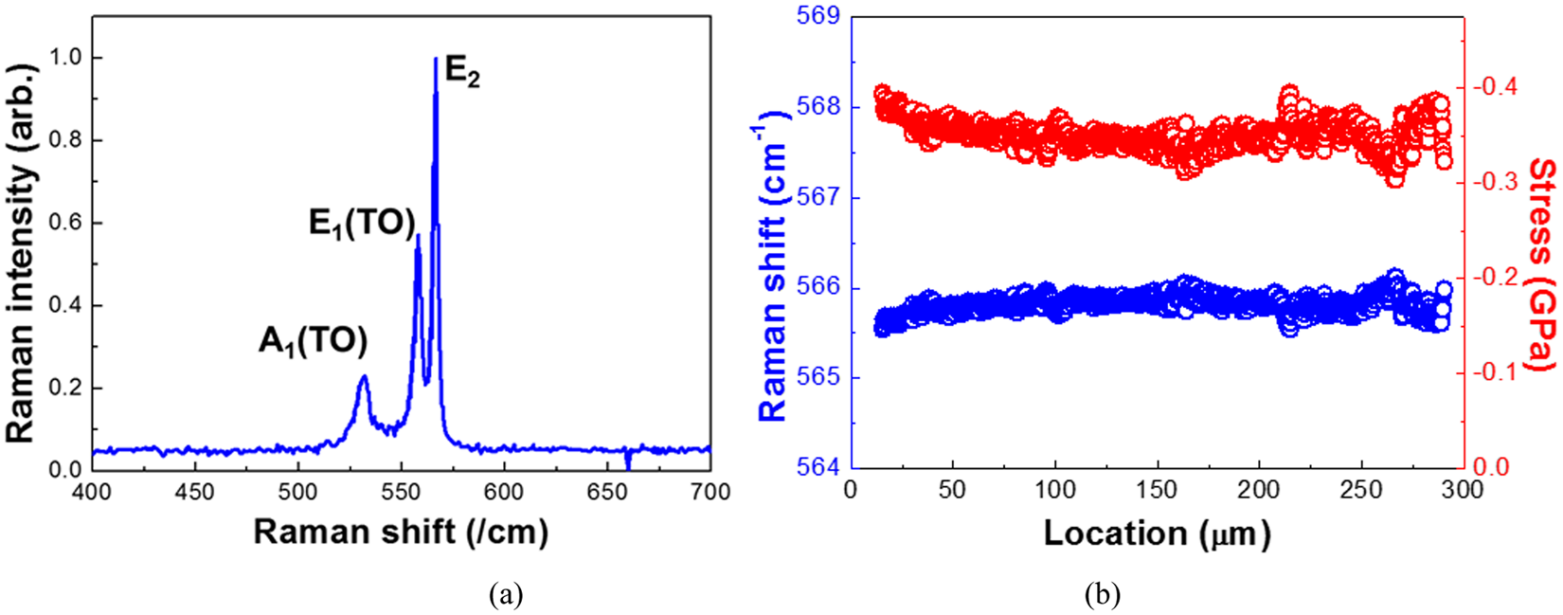
(**a**) Micro Raman spectrum of a freestanding GaN grown from Si substrates; GaN modes are observed at 531.3 cm^−1^ (A_1_), at 558.1 cm^−1^ (E_1_), and 566.5 cm^−1^ (E_2_); (**b**) Micro Raman intensity profiles for E_2_ (high) and calculated stress as a function of the thickness.

Typical stress profiles of the crack-free freestanding GaN crystals grown from Si substrates were studied by micro Raman spectroscopy measurements at room temperature, as shown in Fig. 3(a).

A_1_ (TO), E_1_ (TO) and E_2_ (high) phonon modes can be identified with peaks at 531.3, 558.1 and 566.5 cm^−1^, respectively. The mode frequencies in measured Raman spectra are comparable to the typical 568 cm^−1^ frequency obtained for strain-free bulk GaN^19^. We can easily notice that E_2_ mode frequency has been red-shifted, indicating the residual tensile stress of approaximately −0.35 GPa in freestanding GaN crystals. The residual stress can be calculated using the following equation^20, 21^.

1 $$\sigma =\pm \frac{{\rm{\Delta }}\omega }{{\rm{k}}}(c{m}^{-1}GP{a}^{-1})$$

where σ is the biaxial stress, k is the linear proportionality factor, and ∆ω is the E_2_ phonon peak shift.

Moreover, to determine the strain distribution in freestanding GaN substrate as a function of thickness during HVPE GaN growth, micro Raman spectrum from an edge of a cross-section (X(ZY)$\bar{X}$ geometry) was measured as depicted in Fig. 3(b). Note that the micro Raman intensity for the E_2_ phonon mode has fairly uniform distribution, indicating no significant stress evolution during HVPE GaN growth. Obviously, the residual stress in the GaN layers can be expressed as^22^

2 $${{\rm{\sigma }}}_{{\rm{T}}}={{\rm{\sigma }}}_{{\rm{E}}L}+{{\rm{\sigma }}}_{{\rm{T}}{\rm{H}}}$$

where σ_T_ is the residual stress, σ_TH_ is the thermal stress arising from the difference in the thermal expansion coefficients of the GaN layer and the Si substrate, and σ_EL_ is the elastic stress arising from the lattice mismatch between the GaN layer and the Si substrate at the typical growth temperature. However, the thermal stress, σ_TH_, can be neglected owing to the *in situ* removal of Si substrate, so that the residual stress in HVPE GaN layers is only dependent upon the elastic stress, σ_EL_. Therefore, we can conclude that the tensile stress in HVPE GaN layers observed by the use of Raman measurements is attributed to the elastic stress, indicative of the crystal quality difference between the Ga- and N-face of HVPE GaN.

**Figure 4:**
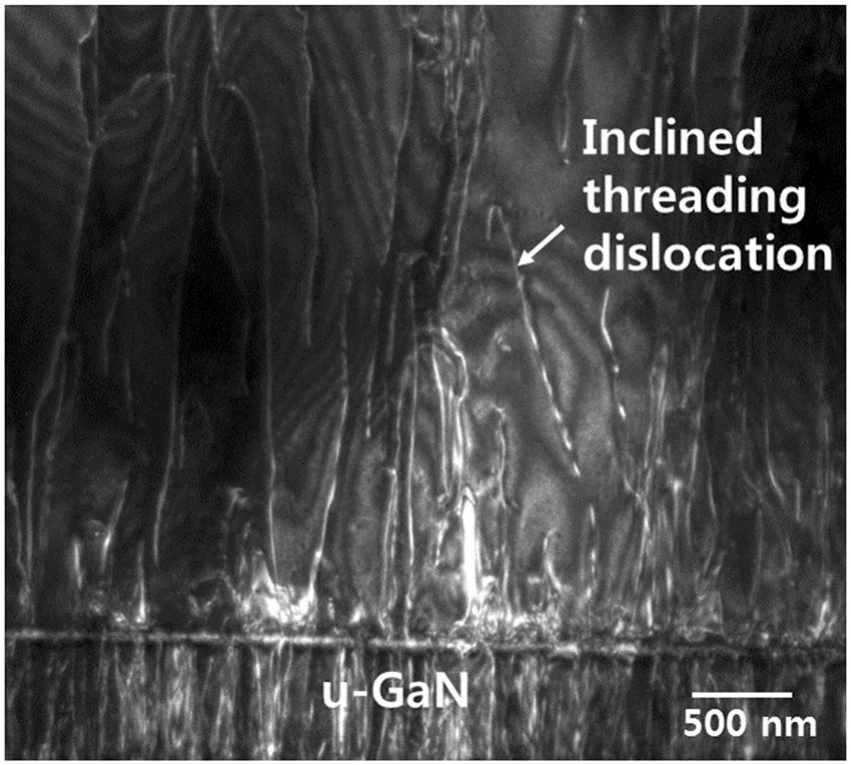
Dark field, cross sectional TEM image of the freestanding GaN crystals grown from Si substrates under diffraction vector g = [11–20].

Figure 4 shows the cross-sectional TEM image of the freestanding GaN crystals grown from Si substrates. It is clearly shown that the dislocations are propagated into growing GaN layers with high inclination angles. Cantu *et. al*. reported that the inclined threading dislocations have a misfit dislocation component, and thus provide the stress relaxation in growing layers^23^.

The stress relaxation of the freestanding GaN crystals was associated with the inclination of threading dislocation lines with respect to the growth direction, generating effective misfit dislocation segments that initially released the stresses in the layers. Therefore, this inclined behaviour of the threading dislocation was intended for the stress relief in the freestanding GaN crystals grown from Si substrates. Additionally, we speculated that this may be related to the lattice mismatch strain between the HVPE GaN and AlGaN/GaN MOCVD buffer layers, by which the local strain environment influences the bending angle of the dislocations^24^. Romanov and Speck reported that the average plastic relaxation due to the inclined threading locations could be quantified as

3 $$\bar{{\rm{\varepsilon }}}=\frac{1}{4}b{\rho }_{TD}\,{\rm{htan}}\alpha $$

where b is the magnitude of the dislocation Burgers vector, ρ_TD_ is the threading dislocation density, h is the film thickness, and α is the inclination angle^25^. The inclination angle in the freestanding GaN layer was approximately 16° from the surface normal, corresponding to an inclination angle of approximately 17.3° in the geometric projection of the dislocation along {1–100}. The density of the threading dislocations and the Burgers vector of (1/3){11–20} are 1 × 10^6^ /cm^2^ and 0.318 nm, respectively. Equation (2) confirms the high strain relaxation of 49.5%, produced by threading dislocation inclination in the 400 µm-thick freestanding GaN. This is in good agreement with the stress relaxation confirmed using the Raman spectrum. We believe that this remarkable strain relaxation prevent the accumulation of tensile stress in the freestanding GaN during growth, thus enabling the growth of the freestanding GaN crystals from Si substrates.

## Conclusions

We investigate the stress evolution of 400-µm-thick freestanding GaN crystals grown on Si by HVPE. We observed that MOCVD AlGaN/AlN buffer layers successfully compensated the stress accumulation on growing HVPE GaN layers. Micro Raman spectroscopy revealed that the stress did not evolve in thick GaN layers during HVPE GaN growth and depended only on the elastic stress arising from the difference in the crystal quality between the Ga- and N-face GaN. Furthermore, TEM analysis showed that the freestanding GaN had inclined threading dislocations, indicative of the stress relief in freestanding GaN layers. We believe that this investigation will inspire the improvement of GaN-based device performances by using freestanding GaN crystals grown from Si substrates.

## Electronic supplementary material

Supplementary information
