# Supplementary material for: The investigation of stress in freestanding GaN crystals grown from Si substrates by HVPE
Source: Sci Rep. 2017 Aug 17;7:8587. doi: 10.1038/s41598-017-08905-y (PMC5561118; doi:10.1038/s41598-017-08905-y)
Supplement: Supplementary file 1 — Supplementary information [file 41598_2017_8905_MOESM1_ESM.docx]

**Supplementary information**

# The investigation of stress in freestanding GaN crystals grown from Si substrates by HVPE

**Moonsang Lee^1*^, Dmitry Mikulik^2^, Mino Yang^3^, and Sungsoo Park^4, 5*^**

^1^Korea Basic Science Institute, Daejeon, 169-148, Republic of Korea

^2^Ecole Polytechnique Fédérale de Lausanne, Laboratory of Semiconductor Materials, Lausanne, 1015, Switzerland

^3^Korea Basic Science Institute, Seoul Center, Seoul, 5, Republic of Korea

^4^ Jeonju University, Department of Science Education, Jeonju, 303, Republic of Korea

^5^Jeonju University, Analytical Laboratory of Advanced Ferroelectric Crystals, Jeonju, 303, Republic of Korea

^*^lms1015@kbsi.re.kr,[sspark@jj.ac.kr](mailto:corresponding.author@email.example)


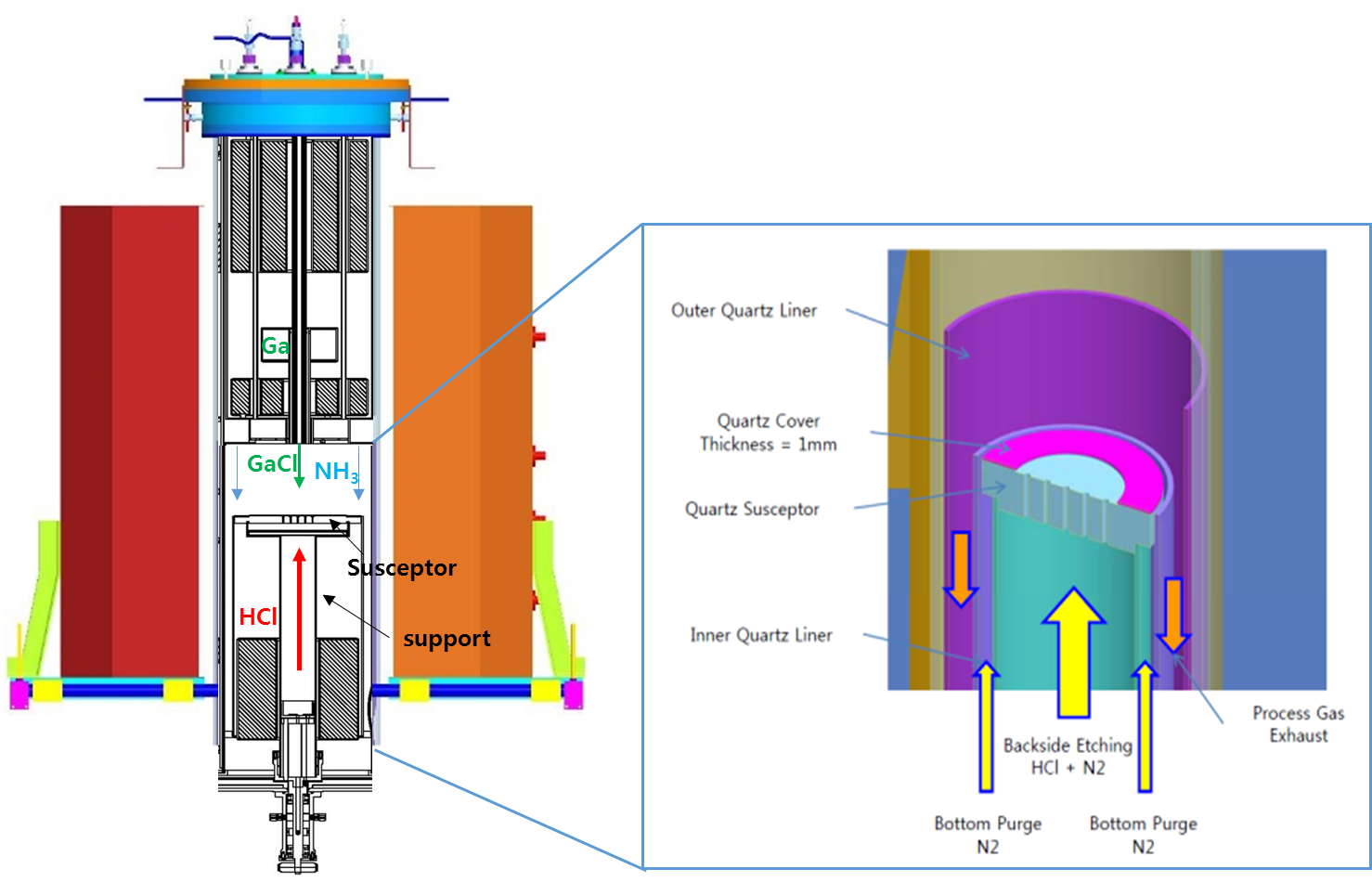


(a)


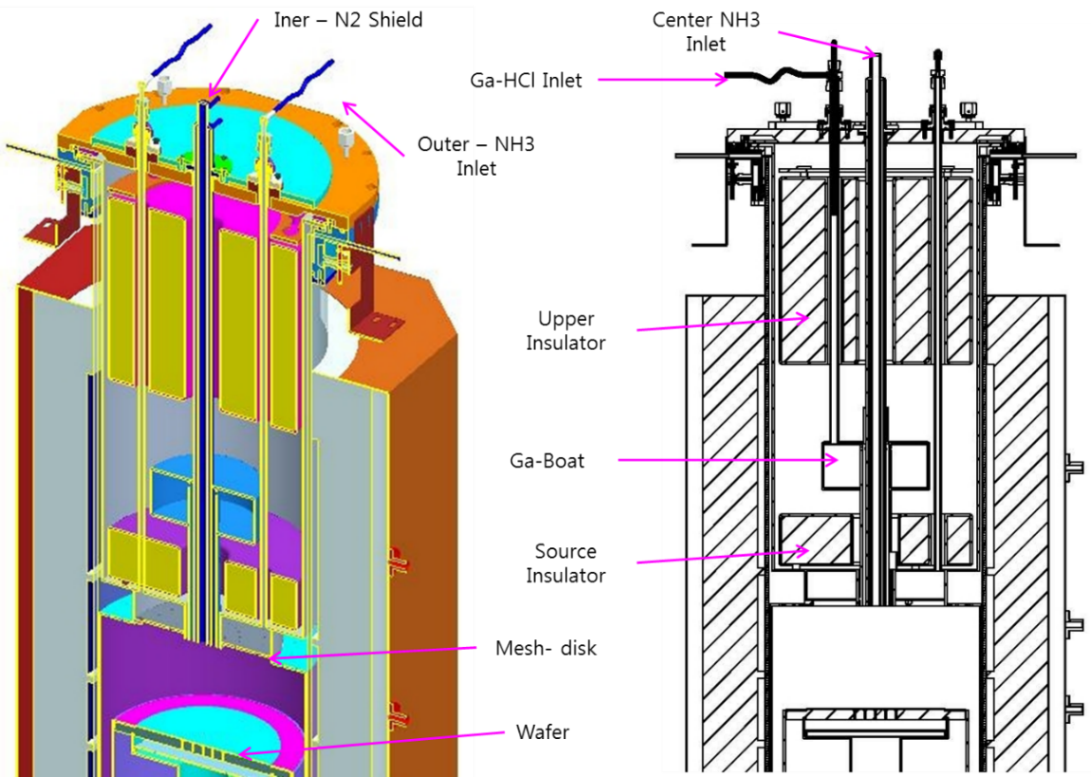


(b)


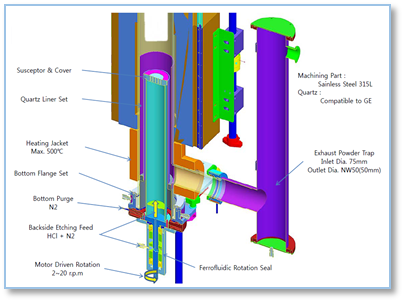


(c)


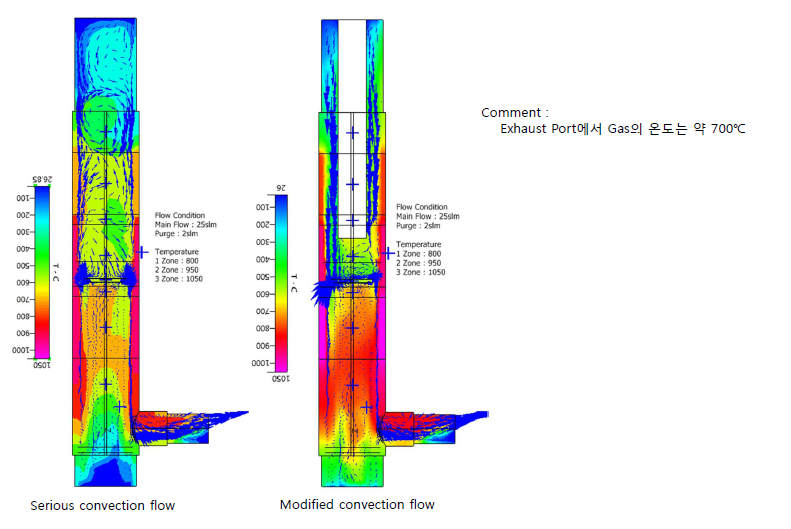


(d)

**Figure S1** Schematic diagram of (a) the modified HVPE reactor design, (b) its gas injection parts, and (c) support parts of the modified HVPE system. (d) numerical analysis of gas and heat stream for the modified HVPE geometry. The computational simulation shows that the convection flow is negligible in the growth zone (zone 3).


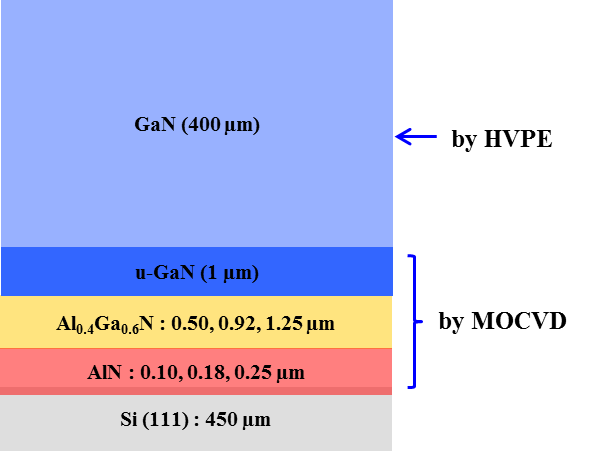


**Figure S2** A schematic diagram for the structures of MOCVD buffer layers. The thickness of AlN and Al_0.4_Ga_0.6_N layers are varied.

The crystal quality of a freestanding GaN crystal grown from Si substrate was confirmed by the micro PL as seen in Fig. 5. Micro PL measurements were carried out using He-Cd laser under excitation at 325 nm at room temperature. In the figure, the dark spots represent a low PL intensity due to non-radiative recombination, implying dislocation sites. This revealed that the dislocation densities were evaluated about 1 × 10^6^ /cm^2^, confirming high crystal quality.


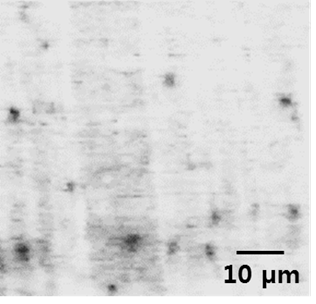


**Figure S3** Micro PL map of a 50 × 50 µm^2^ area of a freestanding GaN crystal with 400 µm in thickness grown from a Si substrate. The bright spots describe high PL emission intensity, and dark spots represent low PL emission intensity, indicating the dislocation sites.


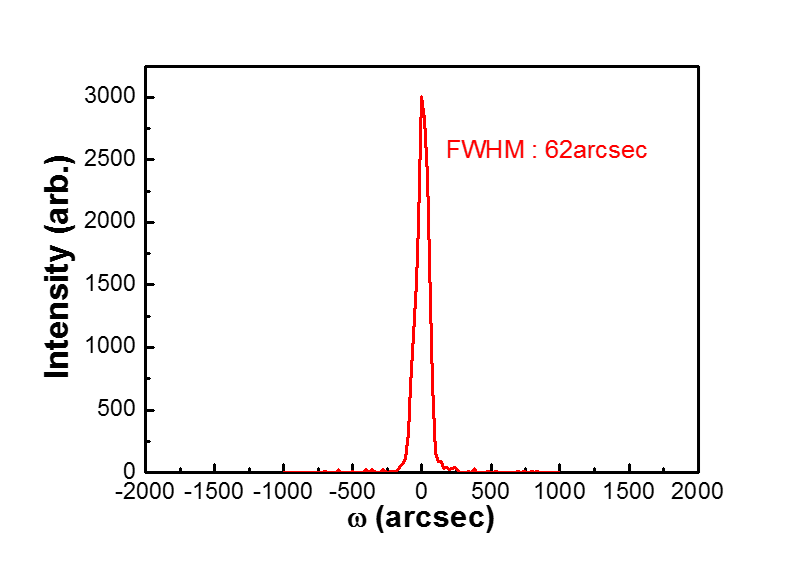


**Figure S4** (0 0 0 2) X-ray rocking curve of a freestanding GaN crystal grown from a Si substrate.

X-ray rocking curves (ω-scan) were measured using the Cu K line (λ = 0.154060 nm) from a Bede D3 system.
